# Associations between demographic, disease related, and treatment pathway related variables and health related quality of life in primary care patients with coronary heart disease

**DOI:** 10.1186/1477-7525-10-78

**Published:** 2012-07-09

**Authors:** Lena Kramer, Oliver Hirsch, Kathrin Schlöβler, Susanne Träger, Erika Baum, Norbert Donner-Banzhoff

**Affiliations:** 1 Department of General Practice/Family Medicine, Philipps University of Marburg, Karl-von-Frisch-Strasse 4, 35043, Marburg, Germany

**Keywords:** Coronary artery disease, Quality of life, Multilevel analyses, Critical pathways

## Abstract

**Background:**

Coronory heart disease (CHD) is a common medical problem worldwide that demands shared care of general practitioners and cardiologists for concerned patients. In order to improve the cooperation between both medical specialists and to optimize evidence-based care, a treatment pathway for patients with CHD was developed and evaluated in a feasibility study according to the recommendation for the development and evaluation of complex interventions of the British Medical Research Council (MRC). In the context of this feasibility study the objective of the present research was to investigate the contributions of different disease related (e.g. prior myocardial infarction), pathway related (e.g. basic medication) and demographic variables on patients` perceived health related quality of life (HRQoL) as a relevant and widely used outcome measure in cardiac populations.

**Methods:**

Data assessing demographic, disease and pathway related variables of CHD patients included in the study were collected in a quasi-experimental design with three study arms (pathway developers, users, control group) via case record forms and questionnaires at baseline and after 6 and 12 (intervention groups), and 9 months (control group), respectively after the initial implementation on GP level. Additionally, at the same measuring points the CHD patients participating in the study were interviewed by phone regarding their perceived HRQoL, measured with the EuroQol EQ-5D as an index-based health questionnaire. Due to the hierarchical structure of the data, we performed cross-sectional and longitudinal linear mixed models to investigate the impact of disease related, pathway related and demographic variables on patients` perceived HRQoL.

**Results:**

Of 334 initially recruited patients with CHD, a total of 290 were included in our analysis. This was an average 13.2% dropout rate from baseline assessment to the 12-month follow-up. At all assessment points, patients` HRQoL was associated with a variety of sociodemographic variables (e.g. gender, employment, education) in each study group, but there was no association with pathway related variables. In both cross-sectional and longitudinal analyses highest HRQoL values in patients were reported in the physician group that had developed the pathway. In the longitudinal analyses there were no significant changes in the reported HRQoL values of the three groups over time.

**Conclusions:**

The found associations between sociodemographic variables and the perceived HRQoL of patients with CHD are in line with other research. As there are no associations of HRQoL with pathway related variables like the basic medication, possible weaknesses in the study design or the choice of outcome have to be considered before planning and conducting an evaluation study according to the MRC recommendations. Additionally, as patients in the developer group reported the highest HRQoL values over time, a higher commitment of the GPs in the developer group can be assumed and should be considered in further research.

## Background

Coronary heart disease (CHD) is worldwide the leading cause for morbidity and mortality in adults [1], although in high income countries the mortality of patients with CHD has decreased in recent years [2,3]. In Germany, prevalence rates in the general population are 6.5% (women) to 9.1% (men) [4]. For affected patients CHD has often significant consequences (e.g. limited functionality, regular medication and monitoring visits) for their entire life. Beside conventional somatic measures like morbidity and mortality, health related quality of life (HRQoL) has become more and more a relevant health outcome to assess the effects of chronic illness on patient’s daily life. In contrast to patients` satisfaction, another widely used outcome measure that was shown in several European studies to be not an adequate outcome due to ceiling effects [5,6], HRQoL is a suitable endpoint in cardiac populations [7,8], also in terms of long-term prognosis [9]. HRQoL is understood as a multidimensional concept representing patient’s subjective perception of his physical, psychological and social health status as well as his general well-being [10]. A common instrument to assess patients HRQoL is the Euroqol EQ-5D [11,12], a generic index-based health questionnaire, which was validated across different diseases, populations and countries [11-14]. In the context of cardiovascular disorders a review by Dyer et al. showed evidence for validity and reliability of the EQ-5D in patients with cardiovascular disease [15]. The EQ-5D index correlates negatively with the severity of cardiac disease. Other factors as age or physical ability (measured in Treadmill exercise time) were shown to have a smaller influence on HRQoL whereas gender had a strong influence [16]. Even after adjustment for depressive symptoms, social support and baseline EQ-5D values, the measured HRQoL of women with CHD was rated lower [17].

To guarantee appropriate care for patients with CHD in primary care, an effective cooperation of general practitioner (GP) and cardiologist with a focus on the GPs´ gatekeeper role is essential. This is of special interest in countries with a social insurance based health care system (SIS; also called “Bismarck” type health care system). In contrast to countries with a national health care system (NHS; also called “Beveridge” type health care system), which is characterized by a strong governmental influence and funding on taxation, SIS are less regulated by institutional standards and funded by non-profit insurance funds. Patients have quasi-universal access to care with only a very limited gate-keeper role of the GP [18]. Thus, competition between medical professionals in the ambulatory sector (primary and secondary care) hinders cooperation. In this context the establishment of shared care plans is – despite their need- difficult to achieve. One approach to facilitate the adaption of shared care is the provision of clinical pathways. Those are multidisciplinary, locally translatable, and involve a stepwise procedure, determined timeframes, and standardized care for a specific clinical problem [19]. In contrast to medical guidelines, which also aim for a standardized patient care, treatment pathways are a more active and more specific form of implementation with a strong bottom-up component [20]. Although the implementation of treatment pathways faces similar problems as the implementation of guidelines [21,22], pathways are supposed to possess higher implementation chances by adapting the guideline recommendations to local conditions [23,24]. However, the effect of local treatment pathways is controversial as not always an implementation benefit by the local adaptation was found [25]. Possible effects of the CHD pathway might be an improvement in medication, more appropriate visits, familiarity with the guideline and exchange between the providers. Due to these assumptions we developed a treatment pathway for patients with CHD in a SIS as most research was yet done in countries with a NHS. As the pathway contained several interacting components and can thus be considered as complex intervention we followed the recommendations regarding the development and evaluation of complex interventions, proposed by the British Medical Research Council (MRC) [26]. According to this iterative model the theory-based development of the complex intervention is followed by exploratory feasibility studies to detect, check and modify potential limitations in the study design before potentially planning and conducting a subsequent evaluation study to assess the intervention’s effectiveness, if possible in a randomized controlled trial (RCT). Within the context of this feasibility study we aimed to investigate the impact of different disease related (e.g. prior myocardial infarction), pathway related (e.g. basic medication) and demographic components on patients` perceived HRQoL as one relevant health outcome in CHD patients.

## Methods

### Development and description of the CHD pathway

The pathway development was performed in cooperation with 14 GPs and 4 cardiologists of the Marburg region, Germany, who were invited by the Department of General Practice/Family Medicine at the University of Marburg. We assumed that by realizing a bottom-up approach [20] physicians` adherence to the recommendations of the treatment pathway would be enhanced. By doing so it was intended to coordinate prescription of drugs and scheduling regular visits with the GP and the cardiologist. The development of the pathway was based on current regional [27] and national care guidelines [28]. Within small working groups moderated by members of our department, plans for monitoring visits (examinations, referral frequency), relevant drugs (including dosage), and documentation forms were developed and brought to a consensus with all participants. To support the implementation of the pathway and to improve the communication and cooperation between GPs and cardiologists in daily practice, we provided the physicians with both a laminated pocket version of the pathway guidelines covering drugs and monitoring visits, and patient treatment logs to list medication and monitoring visits for every patient. An overview of the pocket version of the pathway is given in Additional file 1: Table S1.

### Study design

**Figure 1:**
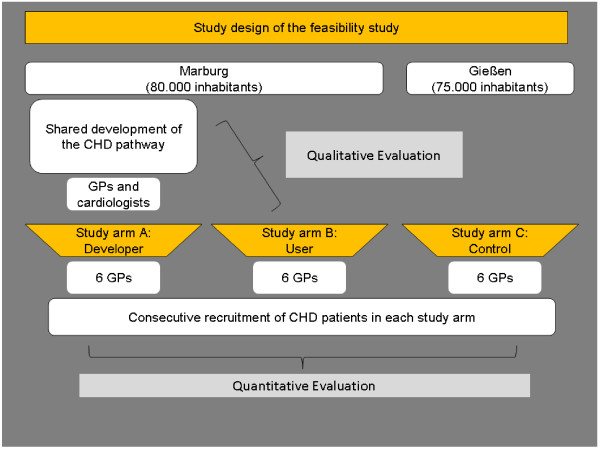
Intended study design.

The study was conducted as a quasi-experimental trial with three study arms. We used GPs as the unit of classification. All practices of the intervention groups (A: pathway developers, B: users) were located in the Marburg region, Germany, whereas the practices of the control group (C) were situated in a neighbouring town. The participating physicians were recruited from the regional physician network of the Department of General Practice/Family Medicine. GPs and cardiologists in both intervention groups received the pathway material. The control group did not receive any pathway material and treated their patients with CHD as usual. GPs got monetary compensation for the recruitment of patients and – if applicable – for their participation in the development of the treatment pathway. All practices were visited by a study nurse to give an introduction in the study flow and documents. Beside the quantitative evaluation of the CHD pathway we conducted semi-structured interviews with the participating GPs to gain further insight into GPs opinion regarding the pathway [29]. An overview of the intended study design is given in Figure 1.

At baseline, data collection for the CHD patients included in the study was performed by the participating GPs in case record forms which contained information about medical investigations, laboratory data like lipid values, liver enzymes and kidney values, medication, referrals to cardiologists, and hospitalizations. In both intervention groups, follow-up assessments with the same case record forms were done at 6 and 12 months. We did not expect remarkable changes in the control group. Therefore, we conducted one follow-up assessment after 9 months. In our analyses we compared the 9 months follow-up data of the control group with both follow-up data of the intervention groups at 6 and 12 months.

Additionally, at the same measuring points patients were asked with standardized questionnaires about their HRQoL and their treatment satisfaction. While at baseline the assessment was paper-pencil based, the follow-up interviews were conducted via phone by independent interviewers. Follow-up intervals of case record form and patients` assessment were identical. Physicians and patients were informed in written form about the study and gave their consent to study participation. Ethic approval for the study was obtained from the Ethics Committee of the Faculty of Medicine at Philipps University Marburg, Germany.

### Data collection

The demographic and disease related variables we included in our analyses comprised patients` age, gender, living with partner, education level, employment, prior myocardial infarction and prior bypass or stent. Regarding the pathway we aimed to investigate the impact of monitoring visits and relevant drugs on patients` HRQoL. As the data for monitoring visits showed no significant variance between groups we excluded this variable from our analyses and focussed on the basic evidence-based medication recommended by the CHD pathway.

Due to the complexity of recommendations regarding drug management, we concentrated on the effects of basic evidence-based medication for CHD on HRQoL. The medication with cholesterol-lowering drugs like statins and antiplatelet agents (e.g. acetylsalicylic acid or clopidogrel) is considered as basic evidence-based therapy in patients with CHD and could be demonstrated as being effective in a variety of studies [30-37]. Based on the prescription of basic evidence-based medication patients were classified as “basic therapy completely achieved” when cholesterol-lowering drugs (statins, fibrates) and antiplatelet agents (ASA, clopidogrel) or alternatively anticoagulant agents (phenprocoumon/warfarin or heparin) were given. If patients received only one drug class they were classified as “cholesterol-lowering drugs achieved” or “antiplatelet/anticoagulant agents achieved”. In spite of their different indication we did not distinguish between antiplatelet and anticoagulant agents as basic evidence-based medication. While anticoagulant agents are recommended for stroke prevention in patients with tachyarrhythmia absoluta and atrial fibrillation or for thrombosis prophylaxis, antiplatelet agents are essential drugs in the treatment of CHD. For patients having an indication for an anticoagulative therapy a combination with antiplatelet drugs is not recommended [30,31]. This recommendation is not valid in comorbid patients having had a myocardial event (infarction, stent implantation) in the past 12 months. In line with the pathway in theses cases a combined medication is indicated. As statins have adverse effects, which might impact the compliance of patients, we also included fibrates as cholesterol-lowering drugs as a basic evidence-based medication.

The primary endpoint of the study was patients` perceived health related quality of life, which was assessed by the Euroqol EQ-5D [11,12]. The EQ-5D describes present-day health status on five dimensions: mobility, self-care, usual activities, pain/discomfort and anxiety/depression. Each dimension is represented by one question with three severity levels (no problems, some or moderate problems and extreme problems). Psychometric qualities are comparable to those of similar instruments like the Short-Form 36 Health Survey Questionnaire (SF-36)[38,39]. The five health dimension items can be used to generate a single index value by applying societal preference weights. These preference weights and an algorithm for calculating the index are available for different countries [40]. According to the recommendations for quality of life analyses in clinical studies we used the preference weights of a European sample [41,42]. The index ranges from 1 (full health) to 0 (dead) with some states being worse than dead (< 0).

### Study sample

According to our study design we asked 18 GPs (6 pathway developers, 6 users, 6 control physicians) to participate in our study and to recruit consecutively patients with CHD. Inclusion criterion was the existence of an ischemic heart disease (I20-I25 of the ICD-10). Patients with currently treated carcinoma, COPD stage III-IV or renal insufficiency stage IV-V were excluded. Sample size calculation was performed with the programme “Sampsize Version 1.0.2” by the Health Services Research Unit of the University of Aberdeen (http://www.abdn.ac.uk/hsru/research/delivery/behaviour/methodological-research). Regarding the EQ-5D Index we aimed to detect a minimum difference of 15 points with a standard deviation of 25. The minimum sample size for each of our intended 18 GPs at a significance level of p = .05, 80% power, and an estimated intracluster correlation coefficient of .10 would be 16 patients. An intracluster correlation of .10 is a conservative estimate [43].

### Data analysis

Baseline characteristics of physicians were compared using the Freeman-Halton extension of Fisher`s exact test because of low numbers in our 2 × 3 tables [44]. The Kruskal-Wallis test was used to examine absolute differences in age.

We analyzed baseline characteristics of patients to identify possible selection bias in the three study arms and performed detailed comparisons between study dropouts and participants.

Cross-tab analyses with χ^2^-tests and standardized residuals were performed using Cramer-V as an effect size. A value of .40 or higher denotes a large effect [45,46]. The Kruskal-Wallis test was used to examine absolute differences in age.

Due to the hierarchical structure of the data (patients nested within physicians) we used cross-sectional and longitudinal linear mixed models with restricted maximum likelihood (REML) estimation to measure associations between predictors and quality of life dependent variables (EQ-5D Index) [47]. This approach is capable of controlling for variations in patient and physician characteristics. Intraclass correlation coefficients (ICC) were calculated for each quality of life variable and assessment point to indicate whether practices were heterogeneous regarding these variables. At an intraclass coefficient of at least .05 a multilevel analysis should be performed [48]. We considered a predictor to be significant if the T statistic of the regression coefficient corresponded to an alpha level of <.05. Tukey`s Least Significant Difference (LSD) test was used to compare adjusted mean differences within significant categorical predictors. Evidence-based medication, the interaction between evidence-based medication and physician group, patients` age, gender, living with partner, education level, employment, prior myocardial infarction and prior bypass or stent were entered as fixed effects. We also tried to integrate them as random effects. Due to the different follow-up assessments between control and intervention groups we applied the last observation carried forward (LOCF) method to impute the missing measurements of the control group at 12 month follow-up with their respective data at 9 month follow-up. Although the use of LOCF has its critics due to its increased potential for bias [49], we thought this approach to be plausible and appropriate as we did not expect change in the control group from the moment of dropout onwards [50]. Patients in the control group had a long history of medical treatment because of CHD and the treatment regiment was not changed during the study period. This has to be considered when interpreting the results [51]. Statistical analysis was performed with SPSS 19.0.

## Results

### Sample characteristics

A total of 20 GPs (8 pathway developers, 6 users, 6 control physicians) participated in the study. Table 1 summarizes the characteristics of the participating GPs.

**Table 1:** Sociodemographic characteristics of the GPs

**Variables**	**Study arm A (developers)**	**Study arm B (users)**	**Study arm C (control)**	**P value**
N	8	6	6	
Mean age (year)	50.8	52.8	51.3	.77
Gender^a^				.06
* female (%)*	4 (50.0)	1 (16.7)	5 (83.3)	
* male (%)*	4 (50.0)	5 (83.3)	1 (16.6)	
Practice location^a^				.87
* town (%)*	4 (50.0)	3 (50.0)	4 (66.6)	
* country (%)*	4 (50.0)	3 (50.0)	2 (22.2)	
Characteristic of the practice^a^				.62
* single practice*	3 (37.5)	2 (33.3)	4 (66.6)	
* group practice*	5 (62.5)	4 (66.6)	2 (33.3.)	

**Figure 2:**
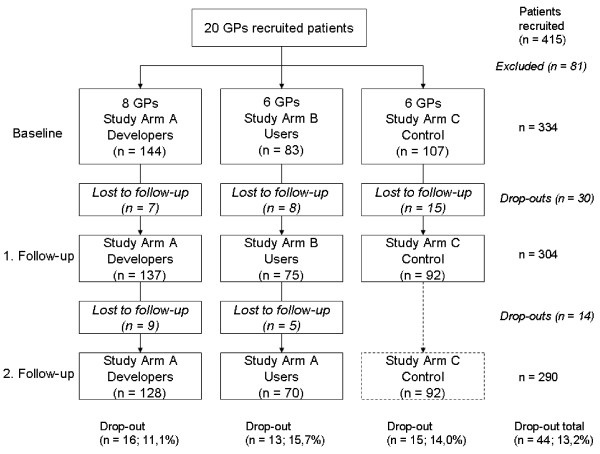
Patient flowchart.

During the recruitment period 415 patients with existing CHD had been invited to participate of whom 334 patients agreed. Of these, 290 patients could be included in the final sample (Figure 2).

The number of patients recruited by each of the 20 GPs ranged between 9 and 24 (mean cluster size 17.1).

As seen in Table 2 most of the participating patients were male (68%), lived with a partner (72%), did not work (80%) and had a mean age of 69 years. The majority (64%) had an education level of 9 or less years, reported prior myocardial infarction (53%) and prior coronary intervention (79%).

**Table 2:** **Sociodemographic and baseline characteristics of the patients**^**a**^

**Variables**	**Study arm A (developers)**	**Study arm B (users)**	**Study arm C (control)**	**P value**
N	128	70	92	
Mean age (year) (SD, range)	68.48 (9.89; 42–91)	68.91 (10.60; 44–89)	70.72 (10.70; 40–94)	.27
Gender^b^				.002
*female (%)*	27 (21.1)	27 (38.6)	38 (41.3)	
* male (%)*	101 (78.9)	43 (61.4)	54 (58.7)	
Living with partner^b^				.02
* yes (%)*	102 (79.7)	49 (71.0)	57 (62.6)	
* no (%)*	26 (20.3)	20 (29.0)	34 (37.4)	
Education level				.25
* 9 or less years*	85 (67.5)	45 (67.2)	55 (62.5)	
* 10 years*	22 (17.5)	6 (9.0)	18 (20.5)	
* 11-13 years*	19 (15.1)	16 (23.9)	15 (17.0)	
Employment				.13
* yes (%)*	30 (23.6)	11 (16.2)	12 (13.3)	
* no (%)*	97 (76.4)	57 (83.8)	78 (86.7)	
Prior myocardial infarction				.46
* yes (%)*	67 (52.3)	42 (60.0)	45 (50.6)	
* no (%)*	61 (47.7)	28 (40.0)	44 (49.4)	
Prior bypass or stent				.16
* yes (%)*	105 (82.0)	58 (84.1)	65 (73.0)	
* no (%)*	23 (18.0)	11 (15.9)	24 (27.0)	

^a^ Numbers may not add up to 290 and percentages may not add up to 100 % due to missing values and rounding.

^b^ Significant difference between groups (α = .05).

There were no differences between the study arms in sociodemographic and baseline characteristics except gender and living with partner. Significantly more patients in study arm A (developers) were male (χ^2^ = 12.09, df = 2, p = .002, Cramer-V = .20) and living with a partner (χ^2^ = 7.77, df = 2, p = .02, Cramer-V = .16), whereas in study arm C (control) women not living with a partner were overrepresented, compared to the other groups.

### Dropout analysis

There was an average 13.2% dropout rate from baseline assessment to the final follow-up assessment (n = 44). The proportion in study arm B (users) was highest with 15.7% (13 out of 83 at baseline), in study arm C (control) 14.0% (15 out of 107) and in study arm A 11.1% (16 out of 144 at baseline) (see Figure 2).

There were no differences between the final sample and dropouts in sociodemographic and baseline characteristics except living with partner (χ^2^ = 4.81, df = 1, p = .03, Cramer-V = .12), employment (χ^2^ = 5.01, df = 1, p = .03, Cramer-V = .12) and prior myocardial infarction (χ^2^ = 5.85, df = 1, p = .02, Cramer-V = .13). Dropouts more often lived without partner, did not have an employment and had no myocardial infarction in the past compared to the final sample. All effect sizes can be considered small.

### Cross sectional analyses

As Table 3 shows, ICCs are > .05, so that multilevel analyses are feasible.

**Table 3:** Means and standard deviations (sd) of EQ-5D index and their respective intraclass correlation coefficients (ICC)

**Group**	**Index t0**	**Index t1**	**Index t2**
Developers	80.59 (16.95)	78.12 (17.29)	77.82 (16.08)
Users	68.74 (20.71)	69.36 (21.04)	66.20 (18.70)
Control	69.94 (20.97)	66.45 (25.39)	66.45 (25.39)
ICC	.129	.089	.100

Table 4 summarizes the results of the multilevel cross sectional analyses for all assessment points. The integration of the predictors as random was not possible because the respective models did not converge.

**Table 4:** Estimated multilevel regression coefficients for fixed effects, their standard errors (in parentheses), and p value of the T statistic for the predictors and the dependent variables at baseline, first and second follow-up

	**Index t0**	**Index t1**	**Index t2**
group	−4.58 (3.08) p = .14	−6.21 (3.05) p = .05*	−7.87 (2.98) p = .01**
medication	−1.72 (3.47) p = .62	−2.66 (3.71) p = .48	−5.50 (3.50) p = .12
group*medication	−0.14 (1.65) p = .93	0.92 (1.74) p = .60	2.10 (1.65) p = .21
myocardial infarction	−3.17 (2.38) p = .19	−3.66 (2.57) p = .16	−5.20 (2.48) p = .04*
bypass/stent	1.80 (3.04) p = .56	−0.93 (3.28) p = .78	1.27 (3.16) p = .69
gender	−2.77 (2.72) p = .31	−9.50 (2.95) p = .001**	−6.45 (2.84) p = .02*
age	0.18 (0.15) p = .21	0.01 (0.16) p = .97	−0.10 (0.15) p = .53
partner	−2.75 (2.71) p = .31	−1.09 (2.93) p = .71	−0.86 (2.82) p = .76
employment	−9.21 (3.73) p = .01**	−1.36 (4.02) p = .74	−1.69 (3.88) p = .66
education	2.17 (1.51) p = .15	4.56 (1.62) p = .005**	3.25 (1.57) p = .04*

* p < .05; ** p < .01.

At baseline there was no significant association between any of the predictors and the EQ-5D-Index except for employment. Patients who were employed had a significantly higher EQ-5D Index (81.76, standard error 3.49) than those who were not employed (72.55, standard error 1.84; p = .01).

At the first follow-up assessment patients in the developer group had a significantly higher EQ-5D Index (77.39, standard error 2.66) than those in the control group (67.66, standard error 2.88; p = .02). There was no significant difference between the user group (70.37, standard error 3.20) and these two groups. Male patients (76.39, standard error 2.04) had a significantly higher EQ-5D Index than females (67.23, standard error 2.67; p = .001). Patients with 11 to 13 years of education (77.35, standard error 3.17) had a significantly higher EQ-5D Index than those with up to 9 or less years of education (67.58, standard error 1.89; p = .004).

At the second follow-up assessment patients in the developer group had a significantly higher EQ-5D Index (77.38, standard error 2.31) than those in the control group (68.35, standard error 2.35) and in the user group (66.45, standard error 2.83; p = .02 and p = .01, respectively). Patients without a previous myocardial infarction had a significantly higher EQ-5D Index (73.00, standard error 2.13) than those who had had a prior myocardial infarction (68.46, standard error 2.01; p = .04). Male patients (73.67, standard error 1.85) had a significantly higher EQ-5D Index than females (67.79, standard error 2.50; p = .02). Patients with 11 to 13 years of education (74.50, standard error 2.98) had a significantly higher EQ-5D Index than those with up to 9 years of education (67.37, standard error 1.68; p = .03).

### Longitudinal analyses

Results of the longitudinal analyses are shown in Table 5. The integration of the predictors as random was not possible because the respective models did not converge.

**Table 5:** Estimated multilevel regression coefficients for fixed effects, their standard errors (in parentheses), and p values of the T statistic for the predictors and the dependent variables in the longitudinal analyses

	**Index**
group	−5.02* (2.09) p = .03
time	−1.34 (1.91) p = .48
group*time	−0.08 (0.94) p = .94
medication	−1.52 (0.85) p = .07
myocardial infarction	−4.39** (1.42) p = .002
bypass/stent	0.15 (1.82) p = .94
gender	−6.45** (1.62) p < .001
age	−0.01 (0.09) p = .90
partner	−1.09 (1.62) p = .50
employment	−3.73 (2.23) p = .09
education	3.33** (0.91) p < .001

* p < .05; ** p < .01

**Figure 3:**
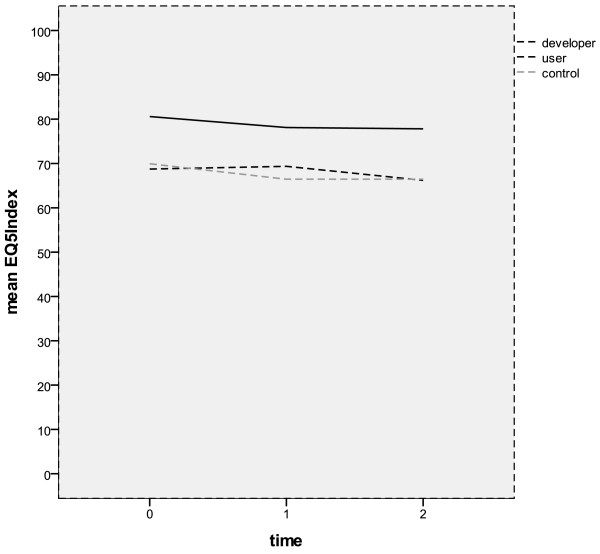
Changes in the EQ-5D Index over the three assessment points.

As seen in Figure 3 the user and the control group had different longitudinal characteristics in the EQ-5D Index over time. At the first follow-up there was a rise in the user group and a decline in the control group. But at the second follow-up, both groups had the same scores again.

Patients with a prior myocardial infarction had a larger decline in the EQ-5D Index than those without myocardial infarction. Women had a larger decline than men in the EQ-5D Index from baseline to first follow-up. Those with 11 to 13 years of education had a rise in the EQ-5D Index from baseline to first follow-up and then a decline while those with up to 9 years and those with 10 years of education had a decline from baseline to first follow-up which then leveled off.

## Discussion

### Main findings

The results of our cross-sectional analyses indicate that at all assessment points, patients` HRQoL was associated with a variety of sociodemographic variables and study group membership whereas there was no association with pathway related variables like the basic evidence-based medication as one major recommendation of the CHD pathway.

The higher values at baseline in employed patients for the EQ-5D Index are in line with the findings gained in populations with other chronic diseases where employment led to higher HRQoL values [52-54]. The EQ-5D Index values at the first and second follow-up rose for patients in the developer group, men and patients with higher education level. At the second follow-up also the absence of a prior myocardial infarction had an impact on patients` HRQoL. Our gender-related findings are coherent with other research as several studies in patients with CHD showed a higher HRQoL for men [55,56]. The reasons for these gender-related differences remain unclear. Possible explanations are that women with CHD are older, have a higher burden of comorbid illnesses and are more often widowed or living alone [16,55]. Also education and the related socioeconomic status are well established determinants for CHD as low education and socioeconomic disadvantage are linked to a higher risk of cardiovascular mortality and morbidity [57].

In the longitudinal analyses EQ-5D Index values declined for women and patients with a prior myocardial infarction over time whereas the gradient for education level showed no linear development. Patients with a prior myocardial infarction can be categorized as having a more severe CHD. In line with our results a review by Dyer and colleagues showed decreased values of the EQ-5D index in patients with severe heart disease [15]. As in our study, women report significantly worse health status after myocardial infarction than men. Compared to both other study groups patients of the developer group at all times had higher values in the EQ-5D index. An explanation for the results of the longitudinal analysis might be the shortness of intervals between the assessment points, so that situational influences could have affected patients` HRQoL ratings.

Higher HRQoL values of patients in the developer group might be due to a higher commitment and deepened preoccupation with the pathway recommendations of those physicians who participated in the pathway development. Grimshaw and colleagues showed in their study that the participation in developing an innovation could enhance the compliance in physicians about 32%, whereas it was improved 22% in non-developers [58]. The appreciation of the developer group regarding their participation in the pathway development could also been shown in our qualitative analysis, where the GPs gave a positive evaluation of their participation and had a high identification with the pathway content [29]. This appraisal of the pathway and its development did not automatically lead to a behaviour change according to the pathway recommendations as the qualitative and quantitative data revealed. In the interviews the GPs reported to have already treated their patients according to the pathway recommendations, but by considering the results of basic drug prescription no difference in prescription behaviour in the three study arms became obvious. Despite these findings patients of GPs who were involved in the development of a complex intervention like the CHD pathway and appreciated this participation showed higher EQ-5D values than patients in the user or control group. This association might be mediated by a modified conduct in the consultation and should be considered in further research.

Since our research question was part of an exploratory feasibility study investigating potential weaknesses in the chosen study design, the results regarding the missing impact of the pathway recommendations (missing variances in monitoring visits, no significance in prescription of basic medication) have to be discussed. Reasons for the found outcome might be up to the intervention itself (e.g. lacking clarity of the pathway material), the implementation of the intervention (e.g. lacking awareness of the pathway in daily practice, prescription routines) or methodological considerations (e.g. choice of endpoint, no randomization), which is partly in line with the findings of our qualitative study [29]. Thus, further feasibility studies should test modifications of the intervention and the study design. In a next step a cluster-randomized evaluation study according to the MRC-model [26] should be conducted to assess the effectiveness of the pathway. Therefore, GPs not familiar with the pathway should be randomized to an intervention group receiving a training regarding the pathway and a control group.

### Study limitations

As a result of the not randomized study design, the representativeness of the data might be limited. Nevertheless, due to the similar baseline characteristics of all study groups – except gender and living with a partner – we assume our results to be valid. We can not exclude a Hawthorne effect because patients in the intervention groups might have noticed that they were treated differently than before and patients in the control group might have noticed they were treated as usual. Another limitation concerns the different follow-up assessments between control and intervention groups and the application of the last observation carried forward method. We thought this principle to be appropriate since we did not expect remarkable changes in the control group over time. Due to practical considerations we included patients with a prevalent CHD while incident cases were neglected. This might have influenced the data. As a consequence of the pre-analysis we did not include monitoring visits in our multi-level analysis. To make a global statement how these pathway recommendations affect patients` HRQoL, reasons for the missing variance in this variable on practice level should be investigated in further research.

## Conclusions

As our findings show, there were associations between different sociodemographic variables, group membership and HRQoL but none with pathway related variables like the recommended basic medication. Thus, further feasibility studies modifying the intervention and/or the study design should be conducted to explore the factors hindering the implementation of the pathway recommendation. Additionally, as patients in the developer group reported the highest HRQoL values over time a larger commitment of the GPs in the developer group can be assumed and should be considered in further research.

## Abbreviations

CHD: Coronary heart disease; HRQoL: Health related quality of life; GP: General practitioner; SIS: Social insurance based health care system; NHS: National health care system; ASA: Acetylsalicylic acid; SF-36: Short-Form 36 Health Survey Questionnaire; ICD-10: International classification of diseases; COPD: Chronic obstructive pulmonary disease; REML: Restricted maximum likelihood; ICC: Intra-class correlation coefficient; LSD: Tukey`s Least Significant Difference.

## Competing interests

The authors declare that they have no competing interests.

## Authors' contributions

NDB and ST contributed to study design. Quantitative data analysis was performed by LK and OH. LK drafted the manuscript. OH, KS, ST, EB and NDB provided critical review on all parts of the manuscript. All authors read and approved the final version of the manuscript.

## Supplementary Material

Additional file 1 Pocket version of the CHD treatment pathway. Click here for file

## References

[B1] Lopez AD Mathers CD Ezzati M Jamison DT Murray CJ Global and regional burden of disease and risk factors, 2001: systematic analysis of population health data Lancet 2006 367 9524 1747 1757 16731270 10.1016/S0140-6736(06)68770-9

[B2] Levi F Lucchini F Negri E La Vecchia C Trends in mortality from cardiovascular and cerebrovascular diseases in Europe and other areas of the world Heart 2002 88 2 119 124 12117828 10.1136/heart.88.2.119 PMC1767229

[B3] Kesteloot H Sans S Kromhout D Dynamics of cardiovascular and all-cause mortality in Western and Eastern Europe between 1970 and 2000 Eur Heart J 2006 27 1 107 113 16204263 10.1093/eurheartj/ehi511

[B4] Lange C Ziese T Daten und Fakten: Ergebnisse der Studie "Gesundheit in Deutschland aktuell 2009" 2010 Berlin: Robert Koch Institut

[B5] Hall JA Horgan TG Stein TS Roter DL Liking in the physician–patient relationship Patient Educ Couns 2002 48 1 69 77 12220752 10.1016/s0738-3991(02)00071-x

[B6] Hirsch O Keller H Albohn-Kuhne C Krones T Donner-Banzhoff N Satisfaction of patients and primary care physicians with shared decision making Eval Health Prof 2010 33 3 321 342 20801975 10.1177/0163278710376662

[B7] Stauber S Schmid JP Saner H Znoj H Saner G Grolimund J Von Känel R Health-Related Quality of Life is Associated with Positive Affect in Patients with Coronary Heart Disease Entering Cardiac Rehabilitation J Clin Psychol Med Settings 2012 published online May 13 10.1007/s10880-012-9311-6 22581108

[B8] Fortin M Dubois M Hudon C Soubhi H Almirall J Multimorbidity and quality of life: A closer look Health Qual Life Outcomes 2007 5 52 17683600 10.1186/1477-7525-5-52 PMC2042974

[B9] Mommersteeg P Denollet J Spertus J Pedersen S Health status as a risk factor in cardiovascular disease: A systematic review of current evidence Am Heart J 2009 157 208 218 19185627 10.1016/j.ahj.2008.09.020

[B10] Radoschewski M Gesundheitsbezogene Lebensqualität - Konzepte und Maße Bundesgesundheitsblatt Gesundheitsforschung Gesundheitsschutz 2000 43 165 189

[B11] Rabin R de Charro F EQ-5D: a measure of health status from the EuroQol Group Ann Med 2001 33 5 337 343 11491192 10.3109/07853890109002087

[B12] EuroQolGroup EuroQol - a new facility for the measurement of health-related quality of life Health Policy 1990 16 199 208 10109801 10.1016/0168-8510(90)90421-9

[B13] Xu R Insinga RP Golden W Hu XH EuroQol (EQ-5D) health utility scores for patients with migraine Qual Life Res 2010 20 4 601 608 21063786 10.1007/s11136-010-9783-5

[B14] Sun S Chen J Johannesson M Kind P Xu L Zhang Y Burstrom K Population health status in China: EQ-5D results, by age, sex and socio-economic status, from the National Health Services Survey 2008 Qual Life Res 2011 20 3 309 320 21042861 10.1007/s11136-010-9762-x PMC3052443

[B15] Dyer MT Goldsmith KA Sharples LS Buxton MJ A review of health utilities using the EQ-5D in studies of cardiovascular disease Health Qual Life Outcomes 2010 8 13 20109189 10.1186/1477-7525-8-13 PMC2824714

[B16] Goldsmith KA Dyer MT Schofield PM Buxton MJ Sharples LD Relationship between the EQ-5D index and measures of clinical outcomes in selected studies of cardiovascular interventions Health Qual Life Outcomes 2009 7 96 19941657 10.1186/1477-7525-7-96 PMC2789057

[B17] Norris CM Spertus JA Jensen L Johnson J Hegadoren KM Ghali WA Sex and gender discrepancies in health-related quality of life outcomes among patients with established coronary artery disease Circ Cardiovasc Qual Outcomes 2008 1 2 123 130 20031799 10.1161/CIRCOUTCOMES.108.793448

[B18] Reibling N Healthcare systems in Europe: towards an incorporation of patient access J Eur Soc Pol 2010 20 5 18

[B19] Kinsman L Rotter T James E Snow P Willis J What is a clinical pathway? Development of a definition to inform the debate BMC Med 2010 8 31 20507550 10.1186/1741-7015-8-31 PMC2893088

[B20] de Stampa M Vedel I Mauriat C Bagaragaza E Routelous C Bergman H Lapointe L Cassou B Ankri J Henrard JC Diagnostic study, design and implementation of an integrated model of care in France: a bottom-up process with continuous leadership Int J Integr Care 2010 10 e034 20216954 PMC2834925

[B21] Vanhaecht K Bollman M Bower K Gallagher C Gardini A Prevalence and use of clinical pathways in 23 countries - an international survey by the European Pathway Association J Integr Care Pathw 2006 10 28 34

[B22] Ovretveit J The future for care pathways J Integr Care Pathw 2010 14 76 78

[B23] Wood DA Kotseva K Connolly S Jennings C Mead A Jones J Holden A De Bacquer D Collier T De Backer G Nurse-coordinated multidisciplinary, family-based cardiovascular disease prevention programme (EUROACTION) for patients with coronary heart disease and asymptomatic individuals at high risk of cardiovascular disease: a paired, cluster-randomised controlled trial Lancet 2008 371 9629 1999 2012 18555911 10.1016/S0140-6736(08)60868-5

[B24] Lelgemann M Ollenschlager G [Evidence based guidelines and clinical pathways: complementation or contradiction?] Internist (Berl) 2006 47 7 690 692–697 16763795 10.1007/s00108-006-1652-5

[B25] Silagy C Weller D Lapsely H Middleton P Shelby-James T Fazekas B The effectivenes of local adaptation of nationally produced clinical practice guidelines Fam Pract 2002 19 3 223 230 11978710 10.1093/fampra/19.3.223

[B26] Craig P Dieppe P Macintyre S Michie S Nazareth I Petticrew M Developing and evaluating complex interventions: the new Medical Research Council guidance BMJ 2008 337 a1655 18824488 10.1136/bmj.a1655 PMC2769032

[B27] Bergert F Braun M Conrad D Ehrenthal K Feßler J Gross J Gundermann K Hesse H Hüttner U Kluthe B Hausärztliche Leitlinie Stabile Angina pectoris und KHK 2006 Köln: Leitliniengruppe Hessen

[B28] Nationale Versorgungsleitlinie Chronische KHK 2007 Köln: Deutscher Ärzte-Verlag

[B29] Kramer L Schlössler K Träger S Donner-Banzhoff N Qualitative evaluation of a local coronary heart disease treatment pathway: practical implications and theoretical framework BMC Fam Pract 2012 13 36 22584032 10.1186/1471-2296-13-36 PMC3489869

[B30] Baigent C Keech A Kearney PM Blackwell L Buck G Pollicino C Kirby A Sourjina T Peto R Collins R Efficacy and safety of cholesterol-lowering treatment: prospective meta-analysis of data from 90,056 participants in 14 randomised trials of statins Lancet 2005 366 9493 1267 1278 16214597 10.1016/S0140-6736(05)67394-1

[B31] Mills EJ Rachlis B Wu P Devereaux PJ Arora P Perri D Primary prevention of cardiovascular mortality and events with statin treatments: a network meta-analysis involving more than 65,000 patients J Am Coll Cardiol 2008 52 22 1769 1781 19022156 10.1016/j.jacc.2008.08.039

[B32] O'Regan C Wu P Arora P Perri D Mills EJ Statin therapy in stroke prevention: a meta-analysis involving 121,000 patients Am J Med 2008 121 1 24 33 18187070 10.1016/j.amjmed.2007.06.033

[B33] Taylor F Ward K Moore TH Burke M Davey Smith G Casas JP Ebrahim S Statins for the primary prevention of cardiovascular disease Cochrane Database Syst Rev 2011 19 1 CD004816 21249663 10.1002/14651858.CD004816.pub4 PMC4164175

[B34] Bhatt DL Role of antiplatelet therapy across the spectrum of patients with coronary artery disease Am J Cardiol 2009 103 3 Suppl 11A 19A 19166708 10.1016/j.amjcard.2008.11.018

[B35] Eidelman RS Hebert PR Weisman SM Hennekens CH An update on aspirin in the primary prevention of cardiovascular disease Arch Intern Med 2003 163 17 2006 2010 14504112 10.1001/archinte.163.17.2006

[B36] Becker RC Meade TW Berger PB Ezekowitz M O'Connor CM Vorchheimer DA Guyatt GH Mark DB Harrington RA The primary and secondary prevention of coronary artery disease: American College of Chest Physicians Evidence-Based Clinical Practice Guidelines (8th Edition) Chest 2008 133 6 Suppl 776S 814S 18574278 10.1378/chest.08-0685

[B37] Patrono C Baigent C Hirsh J Roth G Antiplatelet drugs: American College of Chest Physicians Evidence-Based Clinical Practice Guidelines (8th Edition) Chest 2008 133 6 Suppl 199S 233S 18574266 10.1378/chest.08-0672

[B38] Myers C Wilks D Comparison of Euroqol EQ-5D and SF-36 in patients with chronic fatigue syndrome Qual Life Res 1999 8 1–2 9 16 10457734 10.1023/a:1026459027453

[B39] Wu AW Jacobson KL Frick KD Clark R Revicki DA Freedberg KA Scott-Lennox J Feinberg J Validity and responsiveness of the euroqol as a measure of health-related quality of life in people enrolled in an AIDS clinical trial Qual Life Res 2002 11 3 273 282 12074264 10.1023/a:1015240103565

[B40] Szende A, Oppe M, Devlin N EQ-5D Value Sets: Inventory, Comparative Review and User Guide 2007 Dordrech: Springer

[B41] Greiner W Claes C Schöffski O, Graf v.d. Schulenburg J-M Der EQ-5D der EuroQol-Gruppe Gesundheitsökonomische Evaluationen 2008 Berlin: Springer 403 414

[B42] Greiner W Weijnen T Nieuwenhuizen M Oppe S Badia X Busschbach J Buxton M Dolan P Kind P Krabbe P A single European currency for EQ-5D health states. Results from a six-country study Eur J Health Econ 2003 4 3 222 231 15609189 10.1007/s10198-003-0182-5

[B43] Adams G Gulliford MC Ukoumunne OC Eldridge S Chinn S Campbell MJ Patterns of intra-cluster correlation from primary care research to inform study design and analysis J Clin Epidemiol 2004 57 8 785 794 15485730 10.1016/j.jclinepi.2003.12.013

[B44] Freeman GH Halton JH Note on an exact treatment of contingency, goodness of fit and other problems of significance Biometrika 1951 38 1–2 141 149 14848119

[B45] Ellis PD The essential guide to effect sizes. Statistical power, meta-analysis, and the interpretation of research results 2010 Cambridge: Cambridge University Press

[B46] Grissom RJ Kim JJ Effect sizes for research.A broad practical approach 2005 Mahwah: Lawrence Erlbaum Associates

[B47] Heck RH Thomas SL Tabata LN Multilevel and longitudinal modeling with IBM SPSS 2010 New York: Routledge

[B48] Twisk JWR Applied multilevel analysis. A practical guide 2006 Cambridge: Cambridge University Press

[B49] National Research C The Prevention and Treatment of Missing Data in Clinical Trials 2010 Washington D.C: The National Academies Press 24983040

[B50] Kenward MG Molenberghs G Last observation carried forward: a crystal ball? J Biopharm Stat 2009 19 5 872 888 20183449 10.1080/10543400903105406

[B51] Lancaster GA Dodd S Williamson PR Design and analysis of pilot studies: recommendations for good practice J Eval Clin Pract 2004 10 2 307 312 15189396 10.1111/j..2002.384.doc.x

[B52] Leslie SJ Rysdale J Lee AJ Eteiba H Starkey IR Pell J Denvir MA Unemployment and deprivation are associated with a poorer outcome following percutaneous coronary angioplasty Int J Cardiol 2007 122 2 168 169 17234282 10.1016/j.ijcard.2006.11.052

[B53] Kikuchi H Mifune N Niino M Ohbu S Kira J Kohriyama T Ota K Tanaka M Ochi H Nakane S Impact and characteristics of quality of life in Japanese patients with multiple sclerosis Qual Life Res 2011 20 1 119 131 20700657 10.1007/s11136-010-9725-2

[B54] Lund RS Karlsen TI Hofso D Fredheim JM Roislien J Sandbu R Hjelmesaeth J Employment Is Associated with the Health-Related Quality of Life of Morbidly Obese Persons Obes Surg 2010 21 11 1704 1709 20953731 10.1007/s11695-010-0289-6 PMC3215889

[B55] Duenas M Ramirez C Arana R Failde I Gender differences and determinants of health related quality of life in coronary patients: a follow-up study BMC Cardiovasc Disord 2011 11 24 21619566 10.1186/1471-2261-11-24 PMC3125287

[B56] van Jaarsveld CH Sanderman R Ranchor AV Ormel J van Veldhuisen DJ Kempen GI Gender-specific changes in quality of life following cardiovascular disease: a prospective study J Clin Epidemiol 2002 55 11 1105 1112 12507674 10.1016/s0895-4356(02)00506-1

[B57] Skodova Z Nagyova I van Dijk JP Sudzinova A Vargova H Studencan M Reijneveld SA Socioeconomic differences in psychosocial factors contributing to coronary heart disease: a review J Clin Psychol Med Settings 2008 15 3 204 213 19104965 10.1007/s10880-008-9117-8

[B58] Grimshaw JM Russell IT Effect of clinical guidelines on medical practice: a systematic review of rigorous evaluations Lancet 1993 342 8883 1317 1322 7901634 10.1016/0140-6736(93)92244-n

